# Using an Artificial Neural Bypass to Restore Cortical Control of Rhythmic Movements in a Human with Quadriplegia

**DOI:** 10.1038/srep33807

**Published:** 2016-09-23

**Authors:** Gaurav Sharma, David A. Friedenberg, Nicholas Annetta, Bradley Glenn, Marcie Bockbrader, Connor Majstorovic, Stephanie Domas, W. Jerry Mysiw, Ali Rezai, Chad Bouton

**Affiliations:** 1 Medical Devices and Neuromodulation, Battelle Memorial Institute, 505 King Ave, Columbus, OH, 43201, USA; 2 Advanced Analytics and Health Research, Battelle Memorial Institute, 505 King Ave, Columbus, OH, 4320, USA; 3 Energy Systems, Battelle Memorial Institute, 505 King Ave, Columbus, OH, 43201, USA; 4 Center for Neuromodulation, The Ohio State University, 480 Medical Center Dr, Columbus, OH, 43210, USA; 5 Department of Physical Medicine and Rehabilitation, The Ohio State University, 480 Medical Center Dr, Columbus, OH, 43210, USA; 6 Department of Neurological Surgery, The Ohio State University, 410 W 10th Ave, Columbus, OH, 43210, USA

## Abstract

Neuroprosthetic technology has been used to restore cortical control of discrete (non-rhythmic) hand movements in a paralyzed person. However, cortical control of rhythmic movements which originate in the brain but are coordinated by Central Pattern Generator (CPG) neural networks in the spinal cord has not been demonstrated previously. Here we show a demonstration of an artificial neural bypass technology that decodes cortical activity and emulates spinal cord CPG function allowing volitional rhythmic hand movement. The technology uses a combination of signals recorded from the brain, machine-learning algorithms to decode the signals, a numerical model of CPG network, and a neuromuscular electrical stimulation system to evoke rhythmic movements. Using the neural bypass, a quadriplegic participant was able to initiate, sustain, and switch between rhythmic and discrete finger movements, using his thoughts alone. These results have implications in advancing neuroprosthetic technology to restore complex movements in people living with paralysis.

Neuroprosthetics aim to restore or substitute for a lost function such as motion, hearing, vision, cognition, or memory in patients suffering from neurological disorders. Current neuroprosthetics systems have successfully linked intracortical signals from electrodes in the brain to external devices including a computer cursor, wheelchair and robotic arm^1^,^2^,^3^,^4^,^5^,^6^,^7^,^8^,^9^,^10^,^11^. Recently, a neuroprosthetic bridge that bypassed the injury of the spinal cord was developed to link intracortical signals to a neuromuscular electrical stimulation (NMES) system and enable cortical control of discrete hand movements to a paralyzed person^12^. However, cortical control of more complex rhythmic or oscillatory movements that normally require involvement of the spinal cord has not been demonstrated by current neuroprosthetics technologies.

We hypothesized that one of the reasons for this limitation is the fact that while many rhythmic activities, such as breathing, walking/running, stirring, teeth-brushing, scratching, or playing a musical instrument, are initiated in the brain but they also require further downstream coordination in the spinal cord. Networks of neurons in the spinal cord known as Central Pattern Generators (CPGs) are responsible for producing the rhythmic activities (for a review see ref. 13). CPGs integrate input signal from the brain with sensory feedback from the limbs to produce rhythmic movements. Considerable progress has been made in understanding the behavior of biological CPGs by studying isolated spinal cords in animals (for a review see ref. 14) and using electrical stimulation of the spinal cord to further characterize the properties of the CPGs^15^,^16^,^17^. Researchers have also developed mathematical models of CPGs^18^,^19^ that can generate rhythmic/oscillatory output. These models have been successfully applied in simulation studies of animal and human movements^20^,^21^,^22^,^23^, rehabilitation of paralyzed limbs^24^,^25^ and robotics^26^,^27^,^28^. However, the next step, i.e. the translation from simulation to practical demonstration of real-time cortical control of rhythmic movements in paralyzed limbs has so far been elusive.

To emulate the spinal cord CPG function which facilitates rhythmic movements, we implemented a numerical model for a CPG oscillator and linked it to neural decoding algorithms and a cortically-controlled NMES system. (Supplementary Figure S1 shows the block diagram of the system). We analyzed cortical signals using a wavelet transformation of the data that represented multi-unit activity recorded from a 96-channel multi-electrode array (MEA) implanted in the motor cortex of a human with tetraplegia. We show that the cortical activity patterns for imagined rhythmic flexion/extension movement differ from activity patterns for imagined discrete flexion and extension movements. The wavelet transformed data were input into a Support Vector Machine (SVM)-based neural decoder that could discriminate between brain activity associated with the participant’s desire to perform rhythmic versus discrete movements. Decoded cortical signals were then used to drive the virtual CPG oscillator which in turn controlled the NMES to stimulate the paralyzed muscles and generate movements thereby bypassing the injured spinal cord. Figure 1a,b shows the experimental neural bypass system in use by the participant. Figure 1c,d show examples of raster histograms from one discriminated unit (channel 7 unit 1) with response to attempted thumb movements and the corresponding wavelet processed neural data showing average mean wavelet power (MWP). Using this technology, we have demonstrated for the first time an artificial neural bypass can be used to allow a paralyzed person to perform both discrete and complex rhythmic movements years after injury.

## Results

### CPG model generates rhythmic output

To develop a virtual CPG oscillator capable of generating rhythmic/oscillatory outputs, we carried out simulation studies in MATLAB based on Eqs 1, 2, 3, 4, 5, 6 (see Methods) describing the two-neuron oscillator model as described by Matsuoka^18^ in which the two neurons (the extensor neuron and the flexor neuron) are interconnected in a mutually inhibitive network (Fig. 2a). The parameters used for the model were identified using a genetic algorithm and are listed in Supplementary Table 1. Figure 2b shows the output from the virtual CPG oscillator which was used to control the NMES system, generating stable, rhythmic oscillations at 2.5 Hz using the following parameters: τ_1_ (0.10), τ_2_ (0.20), *β* (8.44), μ_21_ (4.94), μ_12_ (6.00) and, *c* (50.6).

### Neural activity is distinct for imagined rhythmic versus discrete movements

To investigate the changes in neural modulation for imagined rhythmic versus discrete movements we first carried out experiments to analyze neural activity patterns for three trained movements (discrete flexion, discrete extension and rhythmic wiggle) of the thumb and wrist. Motor cortical neural activity was continuously recorded as the participant was cued to imagine one of the three trained movements interleaved with rest periods. Cues were delivered by an animated virtual hand on the computer monitor. MWP was estimated from the cortical neural activity as described in Methods. Representative heat maps of the normalized MWP for each channel overlaid on the physical layout of the array clearly shows the distinct spatial distribution of the neural activity for the imagined rhythmic and discrete movements of the thumb and wrist (Fig. 3).

We also calculated the correlation coefficient between the cue and the MWP on each channel for each motion (Fig. 4). The correlation was significantly different (*p* < 0.05) on 65 out of 96 channels when the participant imagined the rhythmic thumb wiggle compared to when he imagined thumb flexion and on 67 out of 96 channels for imagined rhythmic thumb wiggle compared to imagined thumb extension (Fig. 4a). Similarly, the correlation was significantly different (*p* < 0.05) for imagined wrist wiggle on 67 out of 96 and 74 out of 96 channels compared to imagined wrist flexion and extension respectively (Fig. 4b). Indeed the MWP on some channels was not only highly correlated (correlation coefficient >0.3) for rhythmic movement but was also negatively correlated with either the discrete flexion or extension movement. Figure 4c shows the modulation in MWP on a few representative channels showing distinct modulation for imagined wiggle compared to imagined extension and flexion of the thumb and wrist. It also appears that there are group of channels (for example channel #7, 77, 85, 87, 90) that modulate more when the participant is attempting a rhythmic movement compared to a discrete movement irrespective of the joint (thumb or wrist).

### Distinct neural activity leads to differential cortical control of rhythmic and discrete thumb movements

We next trained the neural decoders to distinguish brain activity between movements. Decoders were trained for a given movement by asking the participant to attempt the three cued thumb/wrist movements interleaved with rest periods. This full set of data was used as input for training and generating the neural decoders. An example of neural decoder training trials is shown in Supplementary Figure S2.

To investigate if the cortically-controlled NMES system could differentially control both discrete flexion/extension movements and more complex, rhythmic movements, we next carried out experiments to test the system’s ability to evoke these movements on cue. For this experiment we focused on analyzing discrete and rhythmic thumb movements as the overhead mounted stereo camera could reliably track movements of the thumb compared to wrist. Test blocks were performed consisting of six trials of each of the three trained movements (discrete thumb flexion, discrete thumb extension and rhythmic thumb wiggle) presented in random order with a rest period between each movement. Cortical activity was continuously decoded using the trained decoders as the participant attempted either the cued movement or rest, as described above.

Neural decoder output corresponding to the rhythmic thumb wiggle was used to trigger the virtual CPG oscillator, which used the identified parameters to generate the oscillatory output used by the NMES for real-time stimulation of alternating extensor and flexor muscles in the participant’s forearm resulting in a rhythmic thumb wiggle. Neural decoder output classified as either a discrete flexion or discrete extension was fed to the NMES for real-time stimulation of muscles controlling either thumb flexion or extension respectively. Representative data, including modulation of MWP (before and after stimulation begins), decoder outputs, and corresponding movement state are shown in Fig. 5a. MWP increases after stimulation begins due to residual stimulation artifact (see Methods). However, since the neural decoders were trained with MWP data from before and during stimulation, the neural decoders were not only able to recognize the correct imagined movement to initiate stimulation but also the participant’s desire to sustain and subsequently terminate the target movement. The participant was able to successfully initiate, sustain, and complete distinct flexion, extension and wiggle movements through his thoughts alone (see Supplementary Video 1). Figure 5b shows the movement state output (as measured by the distance of the thumb from a reference point “sixth finger”, see Methods) generated using finger motion tracking for each movement. Successful discrete flexion/extension and rhythmic wiggle movement attempts can be seen in the figure. An unsuccessful attempt where the participant was unable to correctly initiate rest after a cued flexion can also be seen between 57–60 s. Table 1 shows that performance accuracy, sensitivity, and specificity for the trained movements are high. Using the system, the participant achieved an overall accuracy of 77.4 ± 0.9% (mean ± s.d., *p* < 0.01) across all states of rest, wiggle, extension and flexion. See Supplementary Video 2 showing the participant attempting discrete flexion/extension and rhythmic wrist movements.

## Discussion

Ability to generate rhythmic motion is a fundamental and necessary skill for humans that is needed for a variety of tasks including locomotion (walking/running), breathing, scratching, swimming etc. In this study, we identified and decoded signals recorded from the primary motor cortex of a quadriplegic human to control a virtual CPG oscillator which was then linked to a NMES system to allow volitional control of both rhythmic and discrete movements in the thumb and wrist. Our results show that: (1) stable rhythmic oscillations can be generated using a virtual CPG oscillator, (2) neural activity in the motor cortex is distinct for imagining rhythmic movements compared to discrete movements, (3) neural decoders can be trained to successfully classify imagined rhythmic versus discrete movements, and (4) the decoded neural activity in combination with the CPG oscillator and NMES can differentially enable rhythmic versus discrete movements in a paralyzed joint.

The CPG oscillator model we used to generate oscillatory/rhythmic patterns is based on the biologically-inspired two-neuron oscillator as described by Matsuoka^18^ in which the two neurons (the extensor neuron and the flexor neuron) are interconnected in a mutually inhibitive network (Fig. 2a). A constrained optimization search routine was used in order to identify the parameters for the CPG oscillator that can generate stable oscillations at a desired frequency upon receiving a tonic input from the brain. The CPG oscillator is capable of generating oscillatory output, the amplitude and frequency of which can be independently controlled by tuning the parameters; more specifically the amplitude is positively correlated to the tonic input *c*, the frequency is positively correlated to the adaptation coefficient *β* and negatively correlated to the time constants τ_1_ and τ_2_. For this study, however, we did not attempt to control the amplitude and frequency of oscillation and instead selected parameters that can generate stable oscillations at 2.5 Hz (Fig. 2b). This frequency was selected due to hardware and software limitations as the rate at which the neuromuscular stimulator could be updated was limited to 10 updates/s, and the muscles required approximately 0.2 s of continuous stimulation of the stimulation pattern in order to achieve measureable deflection of the thumb. Similarly the output amplitude of the CPG oscillator was discretized, which was used to trigger the maximum flexion/extension stimulation of the joint in order to achieve the maximum deflection that was physically possible for the thumb or wrist.

We next investigated if neural activity is distinct for imagined rhythmic versus discrete movements. The cortico-spinal tract neurons in layer V of the primary motor cortex (M1) can be divided into two general types - those that connect to spinal interneurons for CPG-driven movement and those that connect directly with muscles and are called the cortico-motoneuronal (CM) cells. Previous work in rhesus monkeys has shown that CM neurons in M1 make monosynaptic connections with motoneurons of shoulder, elbow, and finger muscles for highly skilled movements^29^. Other researchers also correlated CM cell and muscle activity, supporting the idea of a direct connection^30^. These previous findings suggest that different neural modulation patterns should occur in the motor cortex for CPG-driven rhythmic activity compared to slow/discrete type movement of fingers that are controlled through a direct cortico-motoneuronal connection. Indeed we observed significant differences in neural modulation when the study participant was asked to follow visual cues to imagine rhythmic wiggle compared to discrete flexion or extension of the thumb or wrist. Qualitatively, the spatial distribution of neural activity, as shown by the MWP, indicated that the neural activity patterns were not only different (more spatially distributed across the array) but also the neural modulation appeared to be stronger when the participant was asked to imagine rhythmic movement compared to discrete movements (Fig. 3). The increased spatial distribution of neural modulation while imagining higher frequency rhythmic activity is not surprising as it has been shown in humans that during rhythmic activities, such as locomotion, the size of the activated area in the motor cortices changes with walking speed^31^. The measured correlation coefficient between the cue and the corresponding neural activity for each channel on the array indicated that the neural activity was significantly different on a large number of channels when the participant imagined the rhythmic movement compared to discrete flexion/extension movements (Fig. 4a,b). More importantly the neural activity on some channels was not only highly correlated (correlation coefficient >0.3) for rhythmic movement but was also negatively correlated with either discrete flexion or extension movements. In particular the modulation in neural activity on channel #7, 85 and 87 (for thumb) and channel #55, 85, and 87 (for wrist) clearly shows distinct neural modulation when imagining rhythmic wiggle compared to discrete flexion and extension (Fig. 4c). Our observation that there appear to be group of channels that modulate more when the participant is imagining a rhythmic movement compared to a discrete movement irrespective of the joint (thumb or wrist) is consistent with previous findings which indicated that there are group of neurons in the motor cortex that are specifically tuned for CPG-driven rhythmic activity^29^,^30^.

To assess the ability of the system to restore rhythmic and discrete movements, we focused on three thumb movements (rhythmic wiggle, thumb extension and thumb flexion) that were both impaired by the participant’s injury and reactivated by stimulation of forearm muscles. We used SVM-based machine learning algorithms to train and develop neural decoders that discriminate between the neural modulation patterns associated with different imagined movements. SVM is an excellent tool for neural decoding applications and has been used successfully to decode neural activity for neuroprosthetics-related applications^12^,^32^. During the test period, the output of the neural decoders in conjunction with the CPG oscillator was linked to the encoders for NMES of the muscles to enable volitional control of the desired movements in real time. Using the system the participant was able to successfully initiate, sustain and complete the desired rhythmic and discrete movements using his thoughts alone (Fig. 5). The neural decoders not only recognize the correct imagined movement to initiate stimulation but also continue to recognize the participant’s desire to sustain and subsequently terminate the stimulation-induced movement (see Supplementary video 1 showing the participant attempting the three movements along with the corresponding neural activity modulation and decoder outputs). The participant was able to achieve an overall accuracy of 77.4 ± 0.9% as measured by an automated computer-based evaluation of video frames of thumb movements. The overall accuracy is lower than the individual accuracies because it accumulates the errors for each of the three individual movements. Additionally, the computerized system for movement analysis (video classifier) was designed to be able to detect different movements at the same time. For instance, at the beginning of a thumb wiggle, it is difficult to distinguish a wiggle from flexion or extension until the thumb has started moving back and forth. Similarly, a thumb flexion is difficult to distinguish from a wiggle in the first few frames of video until it is clear that the thumb is staying in the flexed position. When conflicts like this occurred, the overall detected move was decided by the video classifier with the highest output and could be error prone for the first few frames of movement. Errors in classification resolved after a few frames of motion as the computerized detection algorithm was able to confirm the presence or absence of oscillatory motions over time.

In conclusion, we showed that a human with quadriplegia can regain a lost function of the spinal cord by using an intracortically controlled neural bypass technology to volitionally control rhythmic and discrete movements. Our system, while invasive, provides an advantage over other strategies that are based on fixed oscillators implemented in the hardware to generate rhythmic movements^21^,^33^,^34^,^35^. These systems lack the important functionality to include the participant’s own intention to control his/her movements. One of the limitations of our current system is the lack of sensory feedback and its integration in the system. While it is known that CPGs can function without sensory feedback, sensory feedback can make the CPGs adaptive to changes, which is important for applications such as locomotion. Our CPG oscillator has the ability to include sensory feedback as an input and we envision incorporating sensory feedback information in future improvements to the system by using sensors placed on joints in the re-animated limb to sense and correct deviations from expected behavior. This will also improve the overall robustness and usability of the system for neuroprosthetics-related applications. Another limitation of our study is that we did not attempt to independently control the amplitude and frequency of rhythmic movements. As mentioned earlier it was due to the limitation of the maximum thumb deflection that can be physically achieved as well as hardware and software limitations. However, by using a biologically-inspired CPG oscillator we have designed a modular system that can be easily adjusted to control both amplitude and frequency by altering the relevant parameters based on the decoded neural modulation.

Our results have significant implications for the development of the next generation of upper or lower limb neuroprosthetic devices that can enable a paralyzed individual to perform tasks that involve both discrete and rhythmic movements. The results also have broader implications in the area of neurorehabilitation, and in particular, hand movement recovery and gait training post stroke and traumatic brain injury. For example, reports in the literature have indicated that rhythmic movements such as those generated during treadmill training can significantly enhance functional motor recovery following spinal cord lesions^36^,^37^ possibly due to changes in the reflex pathways to the pattern generating neurons in the spinal cord^38^. Similarly, evidence points to the idea that physical exercise, including those that involve repetitive or rhythmic regimes, can promote functional recovery after stroke^39^,^40^ possibly due to promotion of neuroplasticity in the motor cortex^41^,^42^. It was also recently shown that using an electroencephalography (EEG)-based BCI training can induce superior associative learning during rehabilitation leading to improved hand functions in chronic stroke patients^43^. It stands to reason therefore, that, a rehabilitation regime involving non-invasive BCI technology driving rhythmic limb movements in stroke patients can further increase the efficacy of neurorehabilitation as it will not only increase the repetition cycles but can also drive neuroplasticity as it will require active learning involving different group of neurons in the motor cortex. The technology presented here demonstrates what is possible in the future and can offer hope for movement restoration and rehabilitation to people living with paralysis worldwide.

## Materials and Methods

Approval for this study was obtained from the US Food and Drug Administration (Investigational Device Exemption) and The Ohio State University Wexner Medical Center Institutional Review Board (Columbus, Ohio). The study met institutional requirements for the conduct of human subjects and was registered on the ClinicalTrials.gov website (Identifier NCT01997125, Date: November 22, 2013). All experiments were performed in accordance with the relevant guidelines and regulations set by the Ohio State University Wexner Medical Center. The participant referenced in this work provided permission for photographs and videos and completed an informed consent process prior to commencement of the study.

### Surgical Procedure

Surgery to implant the electrode array was performed as described in Bouton *et al*.^12^. Briefly, the study participant had complete, non-spastic quadriplegia subsequent to traumatic cervical spine injury sustained 4 years prior while diving. His injury was complete, with an overall neurologic level of C5 AIS A with zone of partial preservation for motor function to C6 bilaterally according to the International Standards for Neurological Classification of Spinal Cord Injury (ISNCSCI)^44^. The patient underwent a left frontoparietal craniotomy for implantation of the microelectrode array into the arm area of his motor cortex. The arm area of motor cortex was identified preoperatively by fusing fMRI activation maps obtained while patient attempted arm movements to the preoperative planning MRI^12^. Intraoperative navigation system was used to plan the craniotomy. Prior to opening the dura, we utilized electrical stimulation to induce contralateral arm movement, confirming the correct location. The array was then implanted into the cortex using a pneumatic inserter. The pedestal was tunneled under the skin to a posterior exit point. Reference wires were placed subdurally as per the manufacturer guidelines.

### System architecture and signal processing

**Figure 1:**
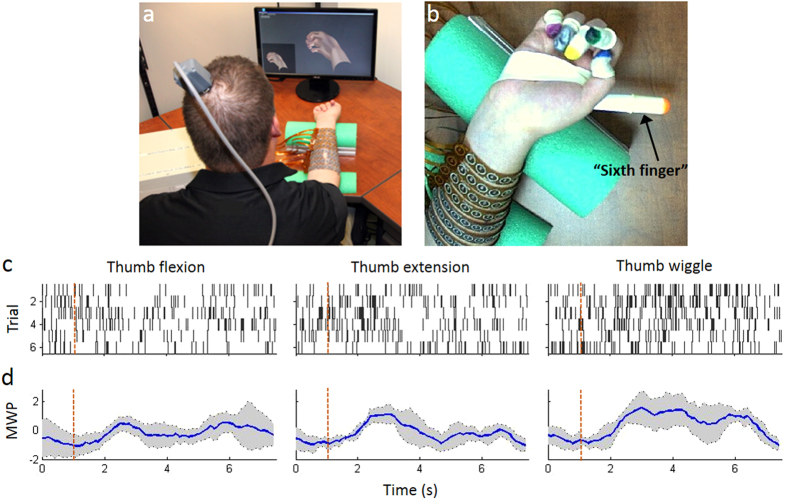
(**a**) Experimental neural bypass system in use with the participant (seated in a wheelchair) in front of a table with a computer monitor. The neuromuscular electrical stimulation sleeve is wrapped around the participant’s forearm; (**b**) Close up view of the participant’s hand showing the colored cots on the fingers. An additional cot was placed on a plastic cylinder extending out past the participant’s thumb (referred to as the “sixth finger”). A stereo camera was positioned above the participant’s hand to track movement in 3 dimensions. The color of the cot on the thumb was used to identify thumb movement and locate it in three-dimensional space with the “sixth finger” serving as the reference point; (**c**) Examples of rasters histograms from one discriminated unit (channel 7 unit 1) with response to attempted thumb movements. The participant was presented with cues to attempt thumb flexion, thumb extension, and rhythmic thumb wiggle. Each cue was presented for a random duration of 2.5–4.5 s followed by a random 2.5–4.5 s rest period. We presented 6 trials of each in random order. Each dot in the raster represents a spike, and each row of spikes represents data from one trial. All trials were aligned on cue presentation (time 1 s, red dashed line). (**d**) Wavelet processed neural data from channel 7 corresponding to the same movements in panel *c* showing the mean wavelet power (MWP) averaged over the trials in dark blue with a confidence interval of ±1 S.D. around the mean shown in grey.

The system consists of three main components: (i) Blackrock microsystem’s Neuroport was used to acquire the neural data being recorded from the implanted array, (ii) A PC running custom neural decoding and encoding algorithms to first analyze the digitized data to determine which motion was being imagined and then encoded back into electrical pulses in order to evoke the desired response from the muscles in the forearm, and (iii) A custom high-definition neuromuscular stimulator which drives the stimulation sleeve wrapped around the forearm of the participant. Figure 1a,b shows the setup of the system and the stimulation sleeve.

Signal collection and processing were performed as described in Bouton *et al*.^12^. Briefly, intracortical signals were recorded and decoded in real-time. A 0.3 Hz 1^st^ order high pass and a 7.5 kHz 3^rd^ order low pass Butterworth analog hardware filter was applied to the data, and each of the 96 channels of the microelectrode array were sampled at a rate of 30 kHz. Multiunit activity (rather than sorted individual units) were used for decoding due to the increased stability of these signals (across the 96 channels) in chronic applications^12^,^45^. A wavelet decomposition was applied to the neural data, using the ‘db4’ wavelet and 11 wavelets scales^46^. The coefficients of scales 3 to 6, corresponding to the multiunit frequency band spanning approximately 234 to 3750 Hz, were then standardized per channel, per scale, by subtracting the mean and dividing by the standard deviation of those scales and channels during an initial rest period at the beginning of the training period. The four scales are then combined by averaging the standardized coefficients for each channel, resulting in 96 values for every 100 ms of data. The resulting values were then used as features, termed mean wavelet power (MWP), to train the decoders and as inputs to the decoders running in real-time.

The signal processing and decoding/control algorithms were all run on a PC using MATLAB (ver 2012a). The digitized data from the Neuroport was processed every 100 ms. Stimulation artifact in the data was detected by looking for a threshold crossing at 500 μV that occurred simultaneously on at least 4 of 12 randomly selected channels. A 2.5 ms window of data around each detected artifact was then removed and adjacent data segments were rejoined to prevent discontinuities. This approach removes a large portion of the artifact, while preserving 87.5% of the original data. Residual artifact remained, however, due to the Neuroport amplifiers recovering from a brief period of saturation after each stimulation pulse, which led to increased MWP. The decoders were trained with data both from the period before and during stimulation to accommodate this residual artifact and allow the user to initiate and sustain the correct movement.

### Participant Sessions and neural decoder training

The study sessions with the participant were typically conducted three times per week, lasting approximately 3–4 hours. Stimulation patterns were first calibrated for the desired movements of the thumb and wrist. Decoders were trained for a given movement by asking the participant to imagine mimicking hand movements cued to him by an animated virtual hand on a computer monitor. The cued movements corresponded to discrete extension, discrete flexion and rhythmic wiggle. Each movement cue was directly followed by a cue to rest and the duration of the movement and rest cues was randomly selected between 2.5 and 4.5 s. The ordering of the movement cues was randomly shuffled so the participant could not anticipate the next cue. The neural decoders were trained in training blocks, each consisting of multiple repetitions of each desired motion. This full set of data was used as input for training a nonlinear Support Vector Machine (SVM) algorithm, to generate a robust set of decoders. A decoder for each motion (against all other motions and rest) was built using a nonlinear Gaussian radial basis function kernel^47^ to process this full set of data and a non-smooth SVM algorithm that uses sparsity optimization to improve performance^32^. During the test period, all decoders ran simultaneously and the decoder with the highest output score above zero was used to drive the NMES.

In order to quantify thumb movements and measure performance, colored finger cots were placed on the participant’s hand during testing. For added sensitivity in detecting movements involving the wrist (used as reference), an additional cot was placed on a plastic cylinder extending out past the participant’s thumb. We refer to this as the “sixth finger” (see Fig. 1b showing test setup). A Bumblebee^®^2 stereo camera (Richmond, Canada) was positioned above the participant’s hand to track movement in 3 dimensions. The color of the cots was used to identify the thumb and locate it in three-dimensional space using a combination of custom code and OpenCV^48^. The location of the thumb relative to the sixth finger was then fed into a classifier that used the same decoding algorithm as the neural decoding but set up to determine thumb movement, termed the video classifier. The video classifier was trained using video data collected at 12 frames/s using scripted stimulation for the moves of interest. Scripted stimulation is a series of predefined stimulation patterns that is used to evoke a known response, without the participant having any control. Overall accuracy for the video classifier was calculated as the fraction of video classifier outputs that match the cue for each frame of video. A permutation test was performed by comparing the observed data to data with shuffled labels^49^. Sensitivity was defined as the proportion of movement cues that were identified correctly and specificity was defined as the proportion of rest cues that were identified correctly. To account for reaction and system lag time the video decoder data was shifted by 1.2 s.

### CPG oscillator

**Figure 2:**
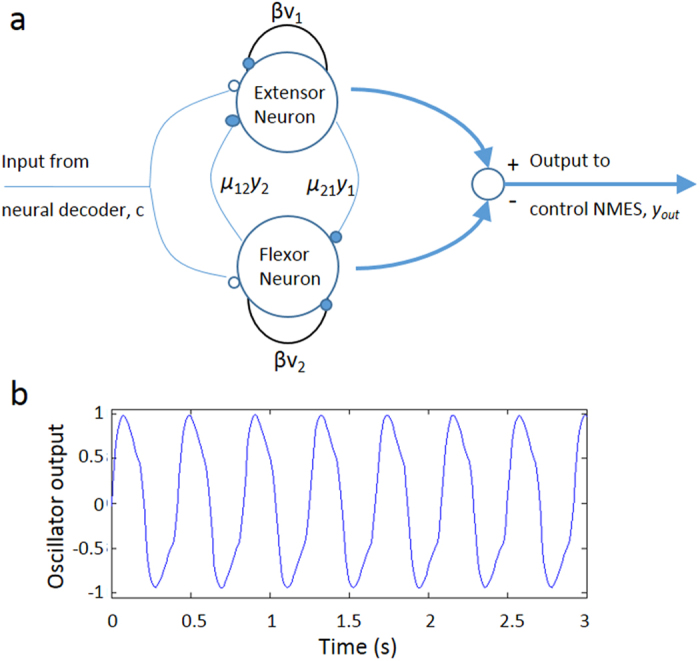
(**a**) A two-neuron oscillator based on the Matsuoka neural model. Neural decoder output corresponding to the rhythmic thumb wiggle was fed as an input to the neuron oscillator model. The output from the oscillator is linked to NMES for stimulating the extensor and flexor muscles controlling the thumb movement; (**b**) Output of the neural oscillator, *y*_out_, showing oscillatory behavior with a frequency of oscillation at 2.5 Hz.

**Figure 3:**
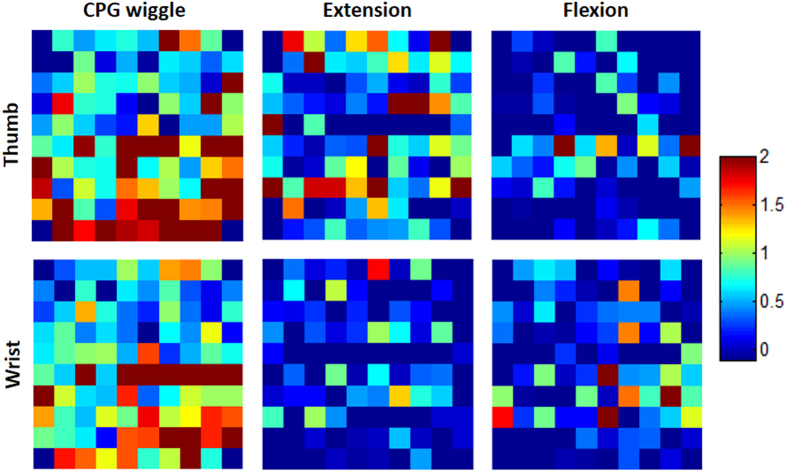
Neural activity is distinct for imagined rhythmic movement. (**a**) Neural firing pattern (as represented by the average normalized MWP power for each move) overlaid on the physical layout of the electrode array shows changes in neural activity while imagining rhythmic/CPG wiggle compared to discrete flexion and extension. The normalized mean wavelet power is in units of standard deviations away from a baseline non-movement period of rest.

**Figure 4:**
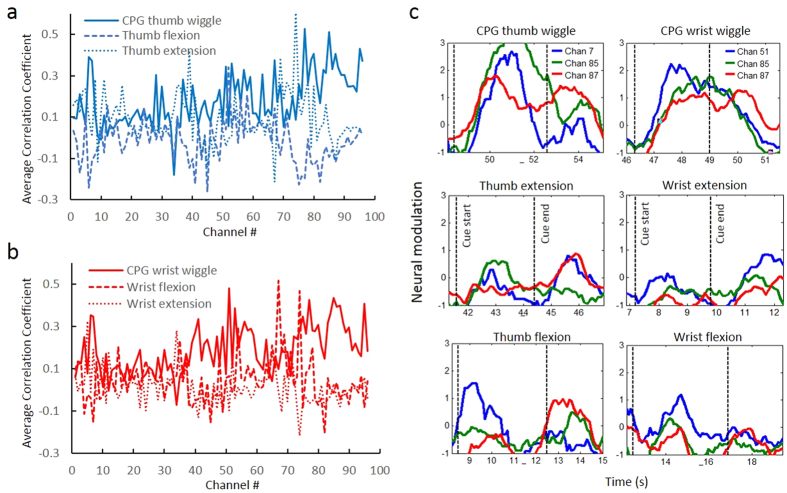
Correlation between neural activity and cue. (**a**,**b**) Correlation coefficient between neural activity and cue vector was calculated for each motion for each array channel and shows the change in modulation levels on certain channels when imagining wiggle compared to flexion and extension. (**c**) Modulation in neural activity on a few representative channels clearly shows distinct neural modulation on these channels when imagining rhythmic wiggle compared to discrete flexion and extension of the thumb and wrist. Data shown ranges from 0.3 seconds before the cue to 2.5 s after the cue ended.

**Figure 5:**
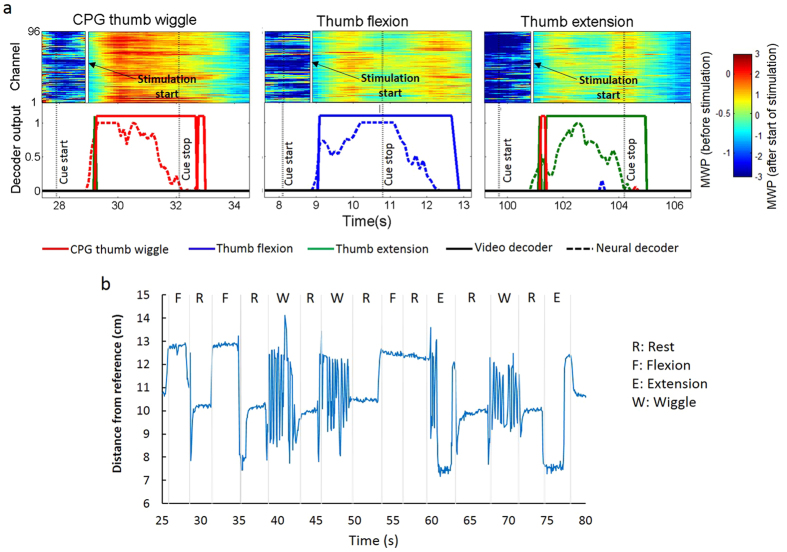
CPG task performance. (**a**) Subfigures show heat maps of MWP representing processed signals from the microelectrode array, neural decoder output scores (dashed line), and physical thumb movements (solid line), as detected by a computer-based video classification algorithm. Of the three simultaneously running decoders, the output score from the one with the highest amplitude greater than zero was used to turn on/off the stimulation. The vertical white line marks when the decoder crosses zero and the stimulation is turned on. MWP increases during stimulation due to residual stimulation artifact (see Methods). The MWP plot is therefore split into two areas with two different color scales (one each for pre- and post-stimulation patterns) to facilitate better visualization of these patterns. The neural decoders are robust to any stimulation induced effects as they not only recognize the correct imagined movement to initiate stimulation but also continue to recognize the participant’s desire to sustain and subsequently terminate the stimulation-induced movement. Trial data shown ranges from 0.3 s before the cue to 2.5 s after the cue ended. A 1 s boxcar filter is applied to the MWP data causing a delay of ~0.5 s. The MWP is in units of standard deviations away from a baseline non-movement period. Only decoder outputs greater than zero are shown for visual clarity; (**b**) shows the results from finger motion tracking for each motion: thumb flexion (F), thumb extension (E), thumb wiggle (W) and a rest (R) in between each motion obtained from the 3D stereo camera mounted overhead. The output is measured as the distance of the finger from a reference point (“sixth finger”, see Methods).

The CPG oscillator to generate oscillatory/rhythmic patterns is based on a two-neuron oscillator model as described by Matsuoka^50^ in which the two neurons (the extensor neuron and the flexor neuron) are interconnected in a mutually inhibitive network (Fig. 2a).

The oscillator network can be expressed by a series of ordinary differential equations (ODEs)^50^,^51^:


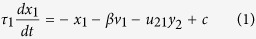



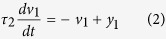



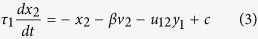



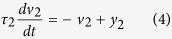



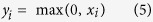



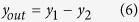


where *x*_*i*_ is the membrane potential of the *i*-th neuron, *v*_*i*_ represents the membrane current, *β* is the adaptive gain for the self-inhibition of neurons, and *c* is the external input. τ_1_ and τ_2_ specify the time constants for adaptation, *μ*_*ij*_ is the weight for the interaction between the neurons, *y*_*i*_ is the neuronal output, and *y*_out_ is the output of the oscillator.

In addition, to produce stable oscillations the following rules must be satisfied^50^:


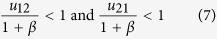



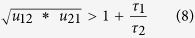


However the set of parameters that can generate stable oscillatory behavior for a desired frequency are not known. Therefore a constrained optimization routine using genetic algorithm was set up using Matlab (Mathworks, Natick, MA) to search for parameters that generate the desired oscillation frequency. There parameters were then used to solve for the ODEs above to generate the CPG output. (see Table S1, Supplementary Information for the list of parameters and corresponding results for frequency of oscillation).

### Stimulation

Neuromuscular electrical stimulation was provided through the use of a custom-made high-definition sleeve as described in Bouton *et al*.^12^. Briefly, the desired movement was evoked by the stimulator providing electrical stimulation through the electrodes on a sleeve wrapped around the participant’s forearm. The sleeve contains 130 electrodes and hydrogel disks (Axelgaard, Fallbrook, CA) of 12 mm diameter. The center-to-center spacing of the electrodes is 22 mm along the long axis of the forearm and 15 mm in the transverse direction. Electrical stimulation was provided in the form of current-controlled, monophasic rectangular pulses of 50 Hz pulse rate and 500 μs pulse width. For each movement, a stimulator calibration was performed to determine the appropriate spatial electrode pattern by using a trial-and-error method. The output score of each movement decoder is between −1 and 1. When the output score of a movement decoder exceeded zero, the system enabled stimulation for that movement. If the output scores of multiple movement decoders exceeded zero simultaneously, then the system enabled the movement with the highest decoder score.

### Code availability

The MATLAB code used to generate the CPG rhythmic patterns from neural signal input can be made available to qualified individuals upon request.

## Additional Information

**How to cite this article**: Sharma, G. *et al*. Using an Artificial Neural Bypass to Restore Cortical Control of Rhythmic Movements in a Human with Quadriplegia. *Sci. Rep.* **6**, 33807; doi: 10.1038/srep33807 (2016).

## Supplementary Material

Supplementary Information

Supplementary Video S1

Supplementary Video S2

## Figures and Tables

**Table 1:** Accuracy, Sensitivity and Specificity of cortically-driven and NMES-generated upper limb movements.

Movement	Thumb Wiggle	Thumb Extension	Thumb Flexion
Accuracy	91.5 + 0.6% (p < 0.01)	90.9 ± 0.6% (p < 0.01)	84.6 ± 0.8% (p < 0.01)
Sensitivity	96.4 ± 1.0%	62.1 ± 2.6%	94.0 ± 1.3%
Specificity	90.6 ± 0.7%	96.4 ± 0.4%	82.9 ± 0.8%
