# Supplementary material for: Using an Artificial Neural Bypass to Restore Cortical Control of Rhythmic Movements in a Human with Quadriplegia
Source: Sci Rep. 2016 Sep 23;6:33807. doi: 10.1038/srep33807 (PMC5034342; doi:10.1038/srep33807)
Supplement: Supplementary Information [file srep33807-s1.pdf]

Using an Artificial Neural Bypass to Restore Cortical Control of Rhythmic Movements in a Human with Quadriplegia

Gaurav Sharma,[1,*] David A. Friedenberg,[2] Nicholas Annetta,[1] Bradley Glenn,[3] Marcie Bockbrader,[4,5] Connor Majstorovic,[1] Stephanie Domas,[1] W. Jerry Mysiw,[4,5] Ali Rezai,[4,6] and Chad Bouton[1,#]

Supporting information

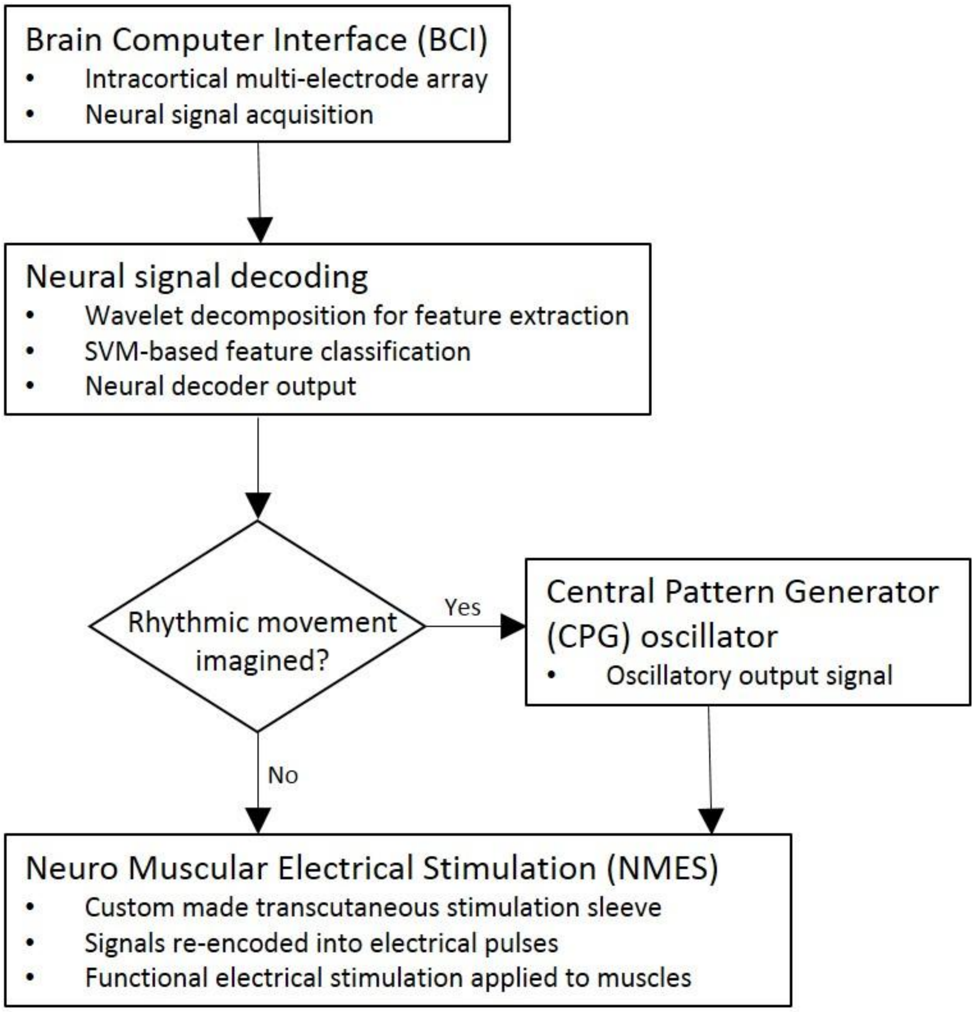

Supplementary Figure S1: Block diagram showing the components of the artificial neural bypass technology to enable cortical control of rhythmic and discrete movements in a human with quadriplegia

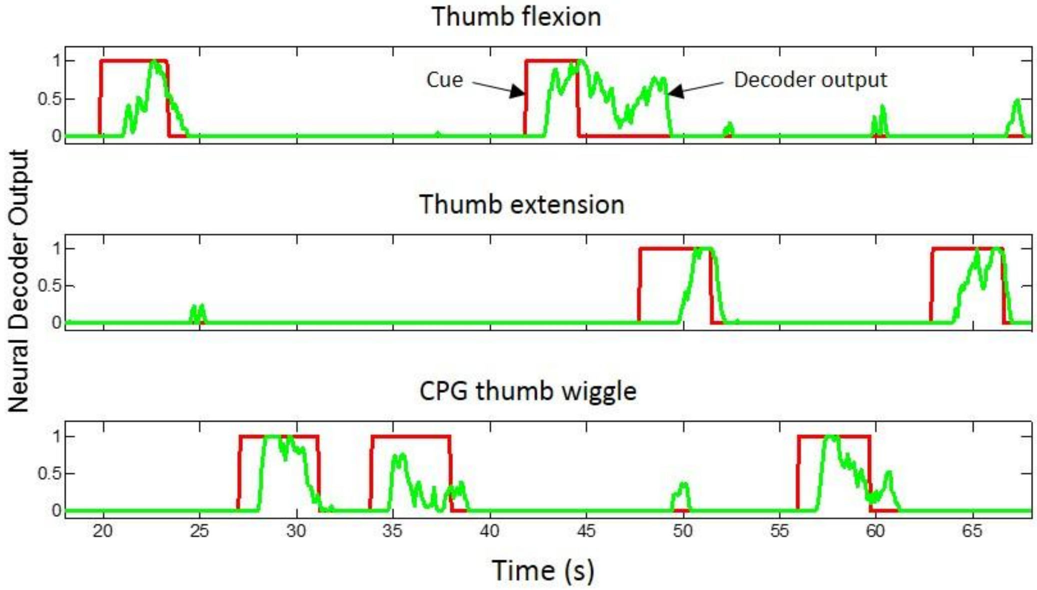

Supplementary Figure S2: Neural decoder output. Data are presented from a subsection of the test block. The red line represents the cue that the participant was trying to match. For a particular movement, when the red line is at one, the user was prompted to imagine that movement. The green line is the decoder output for that move. The decoded movement is 'rest' if none of the decoder outputs are above zero; otherwise, it is determined by the maximum of all the decoder outputs. In this plot the cues have been shifted by 0.8 s to account for reaction and system lag time. Decoder output below zero has been set to zero and output above one has been set to one for visual clarity.

Supplementary Table 1

Frequency (Hz)	τ_1	τ_2	β	μ_{21}	μ_{12}	c
1.1	0.10	0.20	2.50	2.13	3.00	1.10
1.2	0.10	0.20	2.50	2.13	2.00	1.10
1.3	0.10	0.20	2.50	2.09	2.00	1.60
1.4	0.10	0.20	3.00	2.47	2.00	2.35
1.5	0.10	0.20	3.50	2.41	2.00	4.35
1.6	0.10	0.20	3.63	2.41	2.00	6.35
1.7	0.10	0.20	4.13	2.41	2.00	11.60
1.8	0.10	0.20	4.38	2.53	2.00	13.60
1.9	0.10	0.20	4.88	2.66	2.00	15.60
2	0.10	0.20	5.38	2.78	2.00	15.60
2.1	0.10	0.20	5.88	2.78	2.00	15.60
2.2	0.10	0.20	6.38	2.91	2.00	19.60
2.3	0.10	0.20	7.38	3.16	2.00	32.60
2.4	0.10	0.20	7.44	2.94	2.00	48.60
2.5	0.10	0.20	8.44	4.94	6.00	50.60
2.6	0.10	0.20	16.44	12.94	5.50	59.60
2.7	0.10	0.20	16.44	4.94	5.50	59.60
2.8	0.10	0.20	16.44	4.94	9.13	27.60
2.9	0.10	0.20	16.44	5.06	6.88	31.60
3	0.10	0.20	18.44	6.06	6.88	44.60
3.1	0.10	0.20	18.44	4.06	6.88	53.60
3.2	0.10	0.20	18.81	4.06	7.25	69.60
3.3	0.10	0.20	21.06	4.06	8.88	69.60
3.4	0.10	0.20	24.06	4.06	11.63	69.60
3.5	0.10	0.20	26.06	4.06	11.63	78.10
3.6	0.10	0.20	26.06	4.06	9.63	47.10
3.7	0.10	0.20	26.06	4.06	7.63	47.10
3.8	0.10	0.20	26.56	4.06	7.75	83.10
3.9	0.10	0.20	28.56	4.06	7.75	93.10
4	0.10	0.20	29.69	4.06	8.06	61.10
4.1	0.10	0.20	31.69	4.06	8.06	94.10
4.2	0.10	0.20	33.69	4.06	8.06	95.10
4.3	0.10	0.20	34.19	4.06	8.06	47.10
4.4	0.10	0.20	36.19	4.06	8.06	43.10
4.5	0.10	0.20	36.19	20.06	10.31	75.60
4.6	0.10	0.20	36.19	20.06	10.31	80.60
4.7	0.10	0.20	38.19	20.06	10.31	84.60
4.8	0.10	0.20	39.19	20.06	10.31	92.60

4.9	0.10	0.20	39.19	20.06	8.31	88.60
5	0.10	0.20	41.19	22.06	8.31	97.60

Table 1: List of parameters identified by the genetic algorithm for the CPG model

Supplementary Video 1: Rhythmic and discrete thumb movements and corresponding neural activity patterns. This video is a demonstration of the CPG. The subject was randomly presented one of three cues: Thumb Flexion, Thumb Extension, and Thumb Wiggle. The video shows the cue in text at the top along with video of the hand movements and neural activity in a heat map. The units of the heat map are standard deviations above rest.

Supplementary Video 2: Rhythmic and discrete wrist movements This video shows the subject attempting rhythmic and discrete movements of the wrist. The subject was randomly presented one of three cues: Wrist Flexion, Wrist Extension, and Wrist Wiggle. The video shows the cue in text at the top along with video of the hand movements. The small virtual hand on the left of the computer monitor is the "cue hand" which shows the cue while the big hand on the right of the computer monitor is the "feedback hand" which corresponds to the neural decoder output. The deflection of the wrist is small during attempted wrist extension move due to the limitations of extending the wrist beyond what is physically possible. Permission is granted to use the figure with "Battelle" trademark logo in accordance with the *gratis* Open Access License. The "Battelle" trademark is to be acknowledged in future legends.
